# Both Season and Equid Type Affect Endogenous Adrenocorticotropic Hormone Concentrations in Healthy Donkeys, Mules and Hinnies in the United States

**DOI:** 10.3390/ani16020290

**Published:** 2026-01-16

**Authors:** Erin L. Goodrich, Sebastián Gonzalo Llanos-Soto, Renata Ivanek, Toby Pinn-Woodcock, Elisha Frye, Amy Wells, Stephen R. Purdy, Emily Berryhill, Ned J. Place

**Affiliations:** 1 Department of Population Medicine and Diagnostic Sciences, College of Veterinary Medicine, Cornell University, Ithaca, NY 14850, USA; sgl67@cornell.edu (S.G.L.-S.); ri25@cornell.edu (R.I.); tlp52@cornell.edu (T.P.-W.); eab73@cornell.edu (E.F.); amywells@cornell.edu (A.W.); njp27@cornell.edu (N.J.P.); 2 Private Veterinary Practice and Nunoa Project Peru, Belchertown, MA 01007, USA; nunoavet@gmail.com; 3 Department of Medicine and Epidemiology, School of Veterinary Medicine, University of California, Davis, CA 95616, USA; ehberryhill@ucdavis.edu

**Keywords:** donkey, mule, hinny, equid, adrenocorticotropic hormone, ACTH, pituitary pars intermedia dysfunction, Cushing’s disease

## Abstract

Baseline plasma ACTH concentrations are frequently utilized as part of the diagnostic evaluation of equids when PPID is suspected. Baseline ACTH can be impacted by many factors including time of year, i.e., ACTH has generally been found to be elevated during late summer through early autumn in the northern hemisphere. An understanding of ACTH concentrations in healthy equids over the course of a year is useful for the proper interpretation of concentrations in PPID-suspect animals. Previous studies assessing ACTH concentrations in healthy donkeys (*Equus asinus*) and hybrids (*E. asinus* × *E. caballus*) are limited, often utilizing very small numbers, equids from specific and limited geographical regions, limited timeframes or unspecified donkey types (miniature, standard, or mammoth). We aimed to characterize the seasonal variation in baseline ACTH concentrations in healthy miniature donkeys, standard donkeys and hybrids in the United States (US) and to compare those concentrations across these groups. Following outlier removal, 19 standard donkeys (from California (CA), Massachusetts (MA), New York (NY)), 14 miniature donkeys (CA and NY), and 28 hybrids (Texas (TX) and NY) were utilized for analysis. Samples were collected from each equid twice per month from June to November 2019 and once per month from December 2019 through May 2020. The mean ACTH concentration of all equids was higher from mid-August through the end of October compared to the rest of the year (being the highest in the second half of September with the mean (standard deviation) values of 109.6 (52.6), 134.6 (67.4), and 100.8 (189.6) in standard donkeys, miniature donkeys, and hybrids, respectively). Additionally, ACTH concentrations in hybrids were 23% (95% Confidence Interval (CI): 4–38%) and 51% (95% CI: 36–63%) lower than in standard and miniature donkeys, respectively, from mid-August through October. During the rest of the year, hybrids similarly showed 31% (95% CI: 16–43%) and 30% (95% CI: 15–42%) lower ACTH concentrations compared with standard and miniature donkeys, respectively.

## Simple Summary

Donkeys, horses and their hybrid crosses are all susceptible to a degenerative condition called pituitary pars intermedia dysfunction (PPID), or Cushing’s disease, as they age. Correctly diagnosing this disease typically involves recognizing the clinical signs and performing diagnostic testing. A common testing strategy involves measuring baseline plasma adrenocorticotropic hormone (ACTH) concentrations. This study aimed to characterize baseline plasma ACTH concentrations over the course of a year in healthy miniature donkeys, standard donkeys, and hybrids. Mean ACTH values were higher from mid-August through the end of October in all equid groups and both groups of donkeys tended to have higher baseline ACTH concentrations than the hybrids.

## 1. Introduction

PPID is an endocrine disorder that affects horses, donkeys, and their hybrids (mules and hinnies). Despite under-recognition by owners, PPID is thought to occur in around 20% of horses, ponies and donkeys ≥15 years old [1]. The scientific literature describing the epidemiology of PPID in hybrids, however, is very limited [2]. The clinical signs of PPID are similar across aged equid species. Hypertrichosis is considered a pathognomonic clinical feature of advanced PPID in horses, with additional associated signs such as polyuria, polydipsia, muscle wasting, chronic intermittent laminitis and lethargy [3,4]. Identifying donkeys as clinical suspects for PPID is challenging due to their propensity to grow longer, thicker haircoats, and the prolonged duration of spring-time shedding that is typical [5,6]. Additionally, donkeys often have a calm demeanor that may mask detection of lethargy [5,7,8]. Although not specific for the diagnosis of PPID, chronic laminitis may be the most consistent feature of PPID in donkeys, and the United Kingdom Donkey Sanctuary (UK DS) has confirmed PPID in donkeys with clinical and radiographic evidence of laminitis [5,8,9]. Additionally, in a retrospective study of 1444 post mortem examinations of donkeys in the UK DS, 96.3% of those with PPID also had a foot disorder [10]. Identifying chronic laminitis in donkeys can be challenging due to their natural predator avoidance strategies, resulting in a stoic demeanor with subtle pain responses [9]. The literature regarding clinical signs of PPID in mules is very limited but suggests similarities to the other equids [2]. Given the challenges with accurately identifying clinical signs of PPID in donkeys and their hybrids, it is imperative that results of diagnostic testing for this condition are interpreted appropriately.

Measuring baseline plasma ACTH concentration is one component that can assist with making the diagnosis of PPID in equids. Baseline ACTH results are impacted not only by the presence of PPID but also by the time of year, species or type of equid being tested, and other physiological factors [2,8,11,12]. Several publications demonstrate an elevation in baseline plasma ACTH concentration that occurs in both non-PPID and PPID horses during the times of year when daylength is decreasing [11,13,14,15,16,17]. Similarly, this seasonal elevation in ACTH has been detected in donkeys as well [2,11,15,18,19]. Published studies involving ACTH data of hybrid equids are limited. In the most extensive study to date involving any equid hybrids, Gehlen et al. compared baseline ACTH concentrations of donkeys (n = 35) to mules (n = 30) at 3-month intervals from February to November in Germany [2]. That study found that both donkeys and mules experienced a seasonal elevation in ACTH (at the August measurement) and donkeys generally had higher ACTH concentrations than mules at each timepoint [2].

An objective of our present study was to characterize the seasonal variation in plasma ACTH concentrations in healthy miniature donkeys, standard donkeys and hybrids during one year in the contiguous United States (US) and to compare the baseline ACTH concentrations across equid groups [18]. It was hypothesized that (1) healthy miniature and standard donkeys and hybrids would demonstrate higher baseline ACTH concentrations during the periods of the year when daylength was rapidly decreasing, and (2) the healthy donkey groups would display higher baseline ACTH concentrations than the healthy hybrids [2,17,18].

## 2. Materials and Methods

### 2.1. Study Population and Data Collection

A total of 71 apparently healthy individual equids were enrolled in this cohort study, including 21 standard donkeys, 18 miniature donkeys, and 32 mules or hinnies (Table 1). Since mules and hinnies typically cannot be reliably distinguished by observation, they are referred throughout only as hybrids. The donkeys were all either privately owned or part of a university research/teaching herd. The hybrids were residents of a Texas (TX) rescue facility (n = 29) or part of a New York (NY) university research/teaching herd (n = 3). Enrolled standard-sized donkeys resided in California (CA; n = 9), Massachusetts (MA; n = 5), and NY (n = 7), while the enrolled miniature donkeys resided in CA (n = 17) and MA (n = 1).

Enrolled equids were at least 3 years old. The equid’s actual age was obtained based on birthdate when available and otherwise estimated based on dentition [20,21,22]. Individuals ≤15 years were categorized as “Young”, while those >15 years were categorized as “Senior” to allow for statistical analysis of age as a binary variable [23]. All equids were identified either by veterinarians or trained staff as apparently clinically healthy without evidence of PPID (such as chronic laminitis, hypertrichosis or muscle wasting) based on histories and physical examinations [3,5,6]. All equids also had baseline ACTH concentrations assessed within one month of enrollment and were excluded from the study if their pre-enrollment ACTH concentration exceeded 40 pg/mL [2,24]. Enrolled equids were restricted from receiving any daily medications and they did not travel off their farm of origin for more than 7 consecutive days during the study period, to avoid any changes in latitude that may have impacted ACTH concentration [17]. Preventive medicine measures such as vaccination and deworming events were performed based on their veterinarian’s recommendations. Feeding protocols and housing conditions varied according to the discretion of the owner or institution.

Ethical approval for this prospective cohort study was granted by the Cornell University Institutional Animal Care and Use Committee (protocol #2007-0146; approved 24 January 2019).

### 2.2. Specimen Collection and Analysis

All materials necessary for blood collection, storage and shipment were provided to study participants. Samples were collected at multiple timepoints from each equid between June 2019 and May 2020. Blood samples were obtained by venipuncture of the jugular vein using 20 g 1 ½” BD Vacutainer needles (Exel International Medical Products, Saint Petersburg, FL, USA) drawn into plastic EDTA tubes. Blood samples were chilled immediately after collection and centrifuged within 4 h to separate and remove the EDTA plasma to transfer to cryovials. Samples obtained from equids in MA, TX, and CA were frozen at –20 °C prior to being shipped overnight in an insulated Styrofoam cooler with frozen ice packs to the Endocrinology Laboratory at the Cornell Animal Health Diagnostic Center (AHDC) in Ithaca, NY. Specimens collected from donkeys and hybrids in NY were hand-delivered to the AHDC and frozen at −20 °C prior to analysis (within 24 h). EDTA plasma samples were centrifuged again in the Endocrinology Laboratory at 2000× *g* and 4–8 °C for 5–10 min just prior to analysis.

Samples were taken twice monthly (approximately 2 weeks apart, at the beginning and end of the month) from June 2019 to November 2019 and once monthly from December 2019 to May 2020. In months with two blood samples, the first and second sampling events in the month were grouped into the 1st and 2nd batches, respectively (e.g., “September_1–15_” and “September_16–30_” for the two sampling events in September). This distinction in the number of blood sampling events between months was intentional, as it better captures the anticipated seasonal elevation in ACTH concentrations [16]. In addition, samples taken from August_16–31_ through October_16–31_ were classified as ‘Period_mid Aug–late Oct_’, while those taken from all other collection dates (June_1–15_ through August_1–15_ and from November _1–15_ through May_16–31_) were classified as ‘Period_early Nov–early Aug_’. For the purposes of statistical analysis, these time periods were defined a posteriori based on preliminary visual examination of ACTH concentration trends, consistent with previously reported seasonal rises in ACTH seen in donkeys and/or hybrids [2,15,18] and the marginal means observed for donkeys and hybrids over the study period. ACTH concentration was measured using a previously validated chemiluminescence immunoassay (Immulite 2000XPi, Siemens Medical Solutions, Malvern, PA, USA) [17].

#### Statistical Analysis

Descriptive summaries were used to present ACTH concentrations along with age, sex, and sampling location for each animal. For outlier identification and subsequent analyses, standard donkeys and miniature donkeys were treated as separate categories, and hybrids were combined into a single group. Following the outlier screening and exclusion process (detailed below), we conducted statistical analyses to (i) assess the effects of individual characteristics, sampling location, and season on ACTH concentrations, and (ii) estimate summary statistics for ACTH in standard donkeys, miniature donkeys and hybrids. Additionally, for hybrids (n = 28), we estimated ACTH upper reference limits; however, due to small sample sizes, this analysis was not conducted for standard donkeys (n = 19) and miniature donkeys (n = 14) [25]. All statistical procedures were carried out in R (R Core Team, 2020), unless otherwise specified.

### 2.3. Detection and Removal of Outliers and Equids with Insufficient Data

To establish an accurate reference interval for healthy individuals, it is important to exclude outliers, as they can distort calculations and produce misleading ranges, leading to incorrect interpretations of what is considered a normal (or “healthy”) range. Outlier ACTH concentrations by month over the June 2019 through May 2020 study period were identified using the R package *MASS* in R v4.0.3 [26]. This was achieved by applying the robust regression and outlier removal (ROUT) method (q-value = 1%) as described in Motulsky and Brown (2006) to ACTH measurements from each sampling event [27]. The ROUT method was applied separately for the standard donkeys, miniature donkeys, and hybrids. If a single sampling event in months with two samples (i.e., June to November) is identified as an outlier by the ROUT method, then the entire month is considered to have outlier ACTH measurements. This conservative rule was used because the month was the unit of analysis, and it ensured fairness under unequal replication: months with two samples had twice the chance of yielding an outlier compared with single-sample months. Thus, treating any flagged sample as grounds for excluding the entire month prevented systematic bias against months with more measurements. To remove outliers, the following criteria were established:

1. Outlier (suspected of PPID): individual presenting ≥3 outlier ACTH concentrations in months during the ‘Period_mid Aug–late Oct_’ or the ‘Period_early Nov–early Aug_’.
2. Insufficient data: individual with ≥3 months without an ACTH concentration.
3. Apparently healthy: individual not classified as “outlier” or “insufficient data”.

Individuals flagged as “outlier”, or “insufficient data” were removed from further statistical analysis.

### 2.4. Descriptive Statistics

For apparently healthy equids remaining after the removal of outliers and those with insufficient data, we stratified the data by group (standard donkeys, miniature donkeys, and hybrids) and calculated the mean, standard deviation, median, interquartile range, maximum, and minimum ACTH concentrations.

### 2.5. Upper Reference Limit Estimation for Hybrids

Apparently healthy hybrids were included in the estimation of the ACTH upper reference limit using MedCalc v20.023 (MedCalc Software Ltd., Ostend, Belgium) and following the Clinical Laboratory and Standards Institute guidelines [28]. The sample size (<20 individuals) was insufficient to establish upper reference limits for standard and miniature donkeys [25]. ACTH measurement data were Box–Cox power transformed to approximate a normal distribution [29] and the normality was confirmed using the D’Agostino–Pearson test (K^2^ = 1.84, *p* = 0.40), which is considered to offer high specificity in small datasets [30]. The upper reference limit and associated 90% confidence interval (CI) of the transformed data were estimated using a bootstrapped robust method [31]. This robust method has been recommended for sample sizes larger than 20 observations [25]. After estimating the upper reference limit and associated 90% CI, data were backtransformed to their original scale for interpretation.

### 2.6. Linear Mixed-Effects Regression Analysis

Factors associated with ACTH concentrations during each of the ‘Period_mid Aug–late Oct_’ and ‘Period_early Nov–early Aug_’ were identified by building respective linear mixed-effects models in R v4.0.3 using the *glmmTMB* package [32]. To improve model fitness, ACTH measurements were transformed using a natural logarithm (ln) transformation. Variables “Age”, “Sex”, “and “Equid type” were included as fixed effects and variables “Equid ID”, “Sampling date”, and “Location” were included as random effects, considering ACTH concentrations from the same individual and location are likely correlated. Restricted maximum likelihood (REML) was used to fit the linear regression models, and the significance of fixed effects was assessed using Wald’s test. Each fixed effect variable was first evaluated in univariable analysis, which for each period identified a single variable as significant, and thus, the final models for each of the two periods were univariable models; *p*-values and associated 95% confidence intervals (CIs) were estimated using the Kenward–Roger method [33], and significance was interpreted at the *p*-value ≤ 0.05 threshold. Individual variation in ACTH concentration was accounted for by including random intercepts and fixed slopes in the model. A compound-symmetry variance–covariance matrix was used to estimate a correlation pattern from repeated samples. The selection of the variance–covariance matrix structure was based on the Akaike information criterion. The assumptions of normality and homoscedasticity of residuals and random effects were assessed using histograms and scatterplots. To avoid multicollinearity, only numeric predictors that were not correlated (Pearson correlation coefficient ≤ 0.8) were included in the regression analysis [34]. Pairwise differences among categories in a variable were assessed using estimated marginal means and Bonferroni-adjusted post hoc comparisons.

## 3. Results

### 3.1. Data Summary

The process to detect and remove outliers and equids with insufficient data resulted in the removal of 10 of the 71 equids recruited into the study (five individuals from CA, two from NY, and three from TX) with 14 miniature donkeys, 19 standard donkeys, and 28 hybrids remaining for analysis. Table 1 provides information on equids’ location, sex and age (categorized by equid type). Of these equids who remained for analysis, 16 (84%) standard donkeys, 13 (93%) miniature donkeys and 17 (61%) hybrids were ≤15 years old. Sex was distributed between 13 female and 6 (5 castrated) male standard donkeys, 9 female and 5 (4 castrated) male miniature donkeys and 16 female and 12 (0 castrated) male hybrids (Table 1).

**Figure 1:**
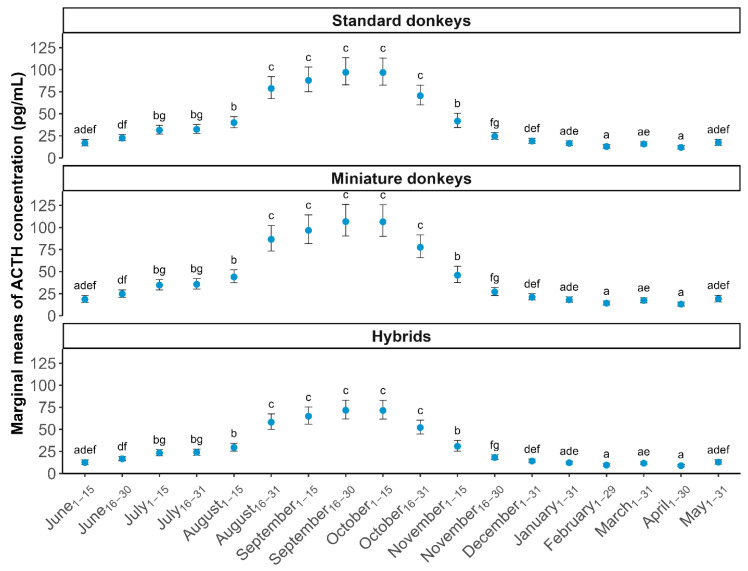
Marginal mean adrenocorticotropin hormone (ACTH) concentrations ± 95% confidence intervals compared across sampling dates for different equid groups: standard donkeys, miniature donkeys, and hybrids. Letters above each estimation indicate statistically significant differences (*p* < 0.05) in ACTH concentration between sampling dates within each equid group.

Table 2 contains summary information on ACTH concentration by sampling the date and age of equids enrolled (after removal of outliers and individuals with insufficient data). The marginal mean ACTH concentrations with 95% CI for each equid species across all sampling dates are shown in Figure 1. Mean ACTH concentrations were elevated from mid-August through the end of October (Period_mid Aug–late Oct_) for all equid groups. The timing of the rise in ACTH was closely aligned between both groups of donkeys and the hybrids (Figure 1).

### 3.2. ACTH Upper Reference Limit for Hybrids

**Figure 2:**
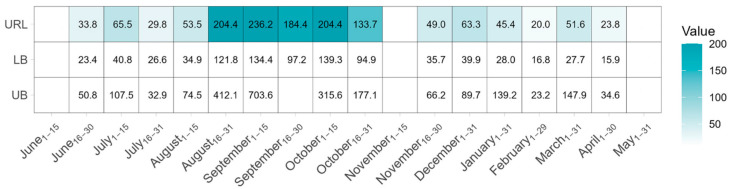
Heatmap showing upper reference limit (URL) values for hybrids across all locations, along with the lower (LB) and upper bounds (UB) of their 90% confidence intervals. Darker shading in the URL cells reflects higher values. The upper reference limit estimation could not be performed during June_1–15_, November_1–15_ and May_1–31_ sampling events due to limited or no observations.

The ACTH upper reference limit and associated 90% CI for hybrids are shown in Figure 2 and Table S1. The upper reference limit estimation could not be performed during the June_1–15_, November_1–15_ and May_1–31_ sampling events due to limited or no observations. The calculated ACTH upper reference limits were found to be elevated during the ‘Period_mid Aug–late Oct_’ compared to the ‘Period_early Nov–early Aug_’. The highest upper reference limit values in the ‘Period_early Nov–early Aug_’ were observed in the July_1–15_ and December_1–31_ sampling events (65.5 pg/mL, 90% CI: 40.8–107.5 pg/mL and 63.3 pg/mL, 90% CI: 39.9–89.7 pg/mL, respectively), while in the ‘Period_mid Aug–late Oct_’, the highest value was recorded in the first half of September (236.2 pg/mL, 90% CI: 134.4–703.6 pg/mL). September_16–30_ had an upper reference limit of 184.4 pg/mL and the associated 90% CI could not be precisely estimated due to the upper bound being >1000 pg/mL. The wide 90% confidence interval observed in this sampling event is likely related to small sample size (n = 28), overall high individual ACTH concentration (interquartile range (IQR): 45.7–82.3 pg/mL), and the presence of an individual with ACTH measurement of 1054 pg/mL (Table 2). Estimations for the month of March were also affected by the highest ACTH measurement (1250 pg/mL) observed among all individuals in the study, causing a large disparity between the mean (70.4 pg/mL) and median (13.4 pg/mL), and resulting in an upper reference limit estimation of 51.6 pg/mL (90% CI: 27.7–147.9 pg/mL).

### 3.3. Regression Analysis

At the univariable level, the mixed-effects linear regression model for the ‘Period_mid Aug–late Oct_’ and ‘Period_early Nov–early Aug_’ included “Equid type” as a significant predictor of ln(ACTH) concentration (Table 3). In both periods, the hybrids group (‘Period_mid Aug–late Oct_’: *p* = 0.02; ‘Period_early Nov–early Aug_’: *p* = 0.000004) showed significantly lower ln(ACTH) concentration compared to standard donkeys. In pairwise comparisons of marginal means, during ‘Period_mid Aug–late Oct_’, hybrids also had lower values than miniature donkeys (*p* = 0.0001), whereas standard and miniature donkeys did not differ (*p* = 0.17). In ‘Period_early Nov–early Aug_’, both standard (*p* < 0.0001) and miniature donkeys (*p* < 0.0001) showed higher ACTH concentrations than hybrids, with no difference between standard and miniature donkeys (*p* = 1.00). For ‘Period_mid Aug–late Oct_’, ACTH concentrations in hybrids were 23.0% (95% CI = 4.4–37.9%) lower compared to standard donkeys and 51.4% (95% CI = 36.2–62.8%) lower compared to miniature donkeys. For ‘Period_early Nov–early Aug_’, we observed a 30.7% (95% CI = 16.1–42.7%) lower ACTH concentration in hybrids compared to standard donkeys and a 29.9% (95% CI = 15.2–41.6%) lower concentration compared to miniature donkeys. No other variables were found to predict ACTH concentration; thus, multivariable models could not be built. Overall, models for both periods presented normally distributed, homoscedastic residuals (Figure S1).

## 4. Discussion

This study demonstrates that seasonal elevations in baseline ACTH concentrations occur in healthy miniature donkeys, standard donkeys and hybrids that reside throughout the contiguous US. The mean baseline ACTH concentrations for all groups of equids were higher during times of rapidly decreasing daylength, from mid-August through the end of October, compared to the rest of the year (Figure 1), which is similar to the seasonal pattern of ACTH observed in healthy US horses [17]. In addition, the larger number of hybrids compared to donkeys enrolled allowed for the estimation of ACTH upper reference limits for hybrids for most months and likewise demonstrated a seasonal variation (Figure 2). Although upper limits were calculated for hybrids, they should still be interpreted with caution due to the relatively small sample size and high variability. It is important to note that upper reference limits are an example of a one-sided reference interval. They differ from clinical decision limits, which were not calculated in this study. Clinical decision limits are calculated in studies comparing known healthy and unhealthy populations and are better utilized for the diagnosis of PPID [35,36]. The upper reference limits determined for healthy hybrids in this study should be used to assess whether further diagnostic testing may be indicated, but they should not be used to definitively distinguish healthy hybrids from PPID-positive hybrids. Since we had fewer than 20 miniature and standard donkeys included in our analysis after outlier detection, we provide summary statistics to guide clinical decision-making, rather than upper limit value estimation for those equid groups.

Previous literature involving ACTH concentration evaluation in healthy donkeys has either not compared the various size categories (miniature, standard, mammoth), or has utilized very small sample sizes, which limited the statistical power [2,11,18,37]. In our statistical analysis, we elected to examine the miniature and standard donkey groups separately due to the low numbers of donkeys enrolled. Our findings do suggest, however, that the standard and miniature donkeys tend to have similar baseline ACTH concentrations throughout the year (Figure 1), but future studies utilizing larger sample sizes and including mammoth donkeys should be performed.

Our study also demonstrated statistically higher ln(ACTH) values in healthy miniature and standard donkeys compared to healthy hybrids during both periods assessed (mid-August to late October versus the rest of the year). When comparing our findings to a horse study that utilized the same sampling strategy and testing laboratory, but enrolled higher numbers of animals across wider geographic latitudes, it is interesting to note that baseline mean ACTH concentrations tend to run higher in donkeys and hybrids than horses throughout the year (Figure 1) [17]. As compared to this horse study of a similar design, our study was limited by relatively small numbers of equids enrolled from a limited geographical range. Future donkey and hybrid ACTH studies should attempt to enroll greater numbers of healthy donkeys and hybrids across latitudinal zones in the US and across age groups, similar to the Pinn-Woodcock et al. horse study, to assess the affects that each of these variables may have on the ACTH baseline values [17]. In our study, the mean baseline ACTH would have been categorized as ‘PPID likely’ using the horse guidelines reported by the Equine Endocrinology Group (EEG) in 4 of 18 (22%) sampling events for standard donkeys, 7/18 (39%) for miniature donkeys and 3/18 (17%) for hybrids [24]. Caution must be taken by clinicians using the EEG guidelines for the interpretation of donkey and hybrid ACTH concentrations. Finally, although sex was not a significant predictor in regression analyses, the unbalanced sex distribution in standard and miniature donkeys (Table 1) may have reduced power to detect sex-specific effects and may have left residual confounding. This imbalance is a limitation of the study.

Baseline ACTH in horses is known to be impacted by several factors, such as stress, trailering, concurrent disease, and exercise [38,39,40,41]. Our study utilized the same outlier detection method as Pinn-Woodcock et al. in their equine ACTH study, thereby minimizing the chances of including PPID-positive donkeys and hybrids [17]. It is worth noting that most of the equids were determined to be apparently clinically healthy by veterinarians but some of the hybrids were assessed by trained staff. In addition, it is known that husbandry factors, body condition and freeze–thaw cycles may impact ACTH concentration, and therefore, are considered potential confounding variables in this study. To the author’s knowledge, there is no scientific literature describing the performance of the thyrotropin-releasing hormone (TRH) stimulation test in healthy donkeys or hybrids; however, Mejia-Pereira et al. have found it to be effective in confirming the diagnosis of PPID in donkeys with clinical signs and elevated baseline ACTH concentrations [42]. Future work should incorporate normal TRH stimulation test results as additional inclusion criteria for enrollment of donkeys and hybrids, which would further minimize the chances of including PPID-positive equids.

## 5. Conclusions

Despite the modest sample size per group and the limited geographical spread, our study shows that baseline ACTH concentrations in healthy donkeys and hybrids vary with season in the US. Baseline ACTH concentrations of healthy donkeys tend to be higher than those of hybrids, and both groups experience seasonal elevation in ACTH concentrations. Future work should attempt to utilize larger sample sizes and incorporate both healthy and PPID-positive equids to establish clinical decision limits, which would further aid in the diagnostic interpretation of values.

## Figures and Tables

**Table 1:** Summary of location, sex, and age characteristics for the enrolled (n = 71) and analyzed (n = 61) apparently healthy equids to characterize adrenocorticotropin hormone (ACTH) variation throughout the year and to assess variables associated with ACTH concentrations. Analyzed equids are those who remained after outliers and individuals with insufficient data were excluded (explained in section ‘Detection and removal of outliers and equids with insufficient data’) and were subjected to statistical analysis to characterize the ACTH variation.

Variables	Enrolled		Analyzed	
	Frequency	%	Frequency	%
Standard donkeys	21	100	19	100
Location				
California	9	43	8	42
Massachusetts	5	24	5	26
New York	7	33	6	32
Sex				
Female	14	67	13	68
Male	7	33	6	32
Age				
Young (≤15 years)	18	86	16	84
Senior (>15 years)	3	14	3	16
Miniature donkeys	18	100	14	100
Location				
California	17	94	13	93
Massachusetts	1	6	1	7
Sex				
Female	12	67	9	64
Male	6	33	5	36
Age				
Young (≤15 years)	17	94	13	93
Senior (>15 years)	1	6	1	7
Hybrids	32	100	28	100
Location				
Texas	29	91	26	93
New York State	3	9	2	7
Sex				
Female	18	56	16	57
Male	14	44	12	43
Age				
Young (≤15 years)	17	53	17	61
Senior (>15 years)	15	47	11	39

**Table 2:** Descriptive statistics for adrenocorticotropin hormone (ACTH) concentrations and age in apparently healthy standard donkeys, miniature donkeys, and hybrids. All equids described in this table were included in the linear mixed-effects regression analysis to identify predictors of ACTH concentration. Additionally, data for hybrids only were used to estimate the ACTH upper reference limits.

Variable	Mean	SD	Median	Min	Q1	Q3	Max	Sample Size
Standard donkeys (n = 19)								
Age (in years)	9.4	5.5	7	3	6	11.5	21	19
ACTH concentration (pg/mL)								
December_1–31_	16.2	7.1	13.6	8.5	12.3	18.5	37.7	19
January_1–31_	14.3	3.4	13.8	8.9	11.9	15.8	22.5	19
February_1–29_	12.6	4.3	11.2	7.2	10.7	14.1	24.2	16
March_1–31_	12.5	3.5	12.7	4.6	11	14.8	18.9	19
April_1–30_	14.5	3.4	14.1	8	11.6	17.5	19.8	19
May_1–31_	18.7	6.1	17.7	7.6	15.8	23.8	27.5	19
June_1–15_	18.9	4	19.1	13.1	15.5	22.2	25.4	14
June_16–30_	29.5	11.6	28.9	15.2	19.4	37	56	19
July_1–15_	38	15.3	33.8	14	28.6	50.6	78	19
July_16–31_	44.6	16.8	41.8	5.3	36.4	53.5	73.3	19
August_1–15_	52.8	18.2	46.7	24	44	64.2	90.1	19
August_16–31_	89.6	39.5	81.1	47.9	63	112.5	207	19
September_1–15_	86.2	31	75.6	38.7	65.8	101.1	144	19
September_16–30_	109.6	52.6	94.4	41.7	68.4	139.5	227	19
October_1–15_	98.4	40	95.6	39.7	64.6	119	179	19
October_16–31_	70.6	45.3	52	16.9	38	91.2	162	19
November_1–15_	53	33.4	43.6	18.5	34.3	62.6	142	19
November_16–30_	24.5	7.2	24.5	12.4	18.7	28.7	39.2	19
Miniature donkeys (n = 14)								
Age (in years)	7.6	6.3	7.5	2	3	8.8	26	14
ACTH concentration (pg/mL)								
December_1–31_	14.5	5.4	13.8	6.1	11.5	15.8	26.9	14
January_1–31_	11.6	3.3	11.8	5	10.1	14.1	16	14
February_1–29_	14.6	14.1	10.8	4	8.7	13.6	61.2	14
March_1–31_	10.9	3.6	11.1	5	8.6	13.2	16.8	14
April_1–30_	12.7	3.3	12.5	6.6	10.9	14.8	18.5	14
May_1–31_	19.7	8	19.4	9.4	14.8	21.9	42.8	14
June_1–15_	17.2	4.2	16	12.5	14	20.6	24.1	13
June_16–30_	34.5	14.3	32.2	18.3	28.4	36.1	79.2	14
July_1–15_	47.4	10.8	46.5	29.9	41.1	54.5	67.7	14
July_16–31_	51.4	14.6	48.5	30.1	42.4	63.8	78.7	14
August_1–15_	82.8	36.6	77.8	27	59.5	96.1	182	14
August_16–31_	87.2	29.4	83.8	51.4	62.2	103.4	144	14
September_1–15_	124.2	56.7	119.5	55.7	90.3	143	287	14
September_16–30_	134.6	67.4	116.5	80.7	88.6	140.5	318	14
October_1–15_	125.6	41.2	114.5	77.2	93	145.8	207	14
October_16–31_	95.3	54	80.7	51.1	64.9	99.2	263	14
November_1–15_	48.7	25.8	36	19.8	29.6	64.6	91.8	14
November_16–30_	28.9	15.6	25	15.7	20.1	32.4	77.1	14
Hybrids (n = 28)								
Age (in years)	13.3	7.4	11.5	3	7	18.2	27	28
ACTH concentration (pg/mL)								
December_1–31_	24.5	15.7	17.9	6.4	14	35.3	71.9	28
January_1–31_	20.5	16.4	15.5	9.1	13.1	19.5	87.7	28
February_1–29_	11.7	4.1	11.1	5.2	9	14.5	21	28
March_1–31_	70.4	239.7	13.4	7.6	12.5	16.8	1250	28
April_1–30_	9.7	7.1	7.3	2	5.8	12.1	37.9	27
May_1–31_	14.4	2.5	14.4	12.6	13.5	15.2	16.1	2
June_1–15_	ND	ND	ND	ND	ND	ND	ND	0
June_16–30_	14.5	8.8	12.4	6.2	9	16.3	47.9	28
July_1–15_	24	18.7	16	4.2	12.4	24.3	69.6	28
July_16–31_	18.6	6.1	18.8	7.4	13.9	23.8	28.8	28
August_1–15_	23.6	18.7	19	2.2	14.5	25.4	102	28
August_16–31_	76.5	97.5	54.6	23	35.4	79.1	553	28
September_1–15_	94.6	169.2	58	28.3	36.8	90.9	946	28
September_16–30_	100.8	189.6	53.2	35.1	45.7	82.3	1054	28
October_1–15_	82	54.2	66.4	31	47.9	91.5	290	28
October_16–31_	63.4	33.1	50.8	17.7	43.1	82.1	145	28
November_1–15_	23.6	1.6	23.6	22.5	23	24.1	24.7	2
November_16–30_	21.7	12.6	17.9	6.6	14.5	25	61.1	28

SD: Standard deviation, Min: minimum, Q1: first quartile, Q3: third quartile, Max: maximum. ACTH: Adrenocorticotropin hormone. ND: No data available.

**Table 3:** Significant predictors for ln-transformed adrenocorticotropin hormone (ACTH) measurements in enrolled apparently healthy equids (n = 61) based on the univariable mixed-effects linear regression analysis conducted separately for the Period_mid Aug–late Oct_ (Aug_16–31_ to Oct_16–31_) and Period_early Nov–early Aug_ (Jun_1–15_ to Aug_1–15_ and Nov_1–15_ to May_1–31_) periods. “Equid Type”, “Sampling date”, and “Location” were considered random effects in the model. The models assumed a compound-symmetry variance–covariance correlation matrix for the random effects. Reference level for factor variable “Equid Type” was “Standard donkeys”.

Variables	Fixed Effect Coefficient	Standard Error	*p*-Value	95% Confidence Interval	
Period_mid Aug–late Oct_ (Aug_16–31_ to Oct_16–31_)					
Type					
Intercept	4.4	0.10	-	-	-
Miniature donkeys	0.25	0.13	0.05	−0.005	0.51
Hybrids	−0.26	0.11	0.02	−0.48	−0.04
Period_early Nov–early Aug_ (Jun_1–15_ to Aug_1–15_ and Nov_1–15_ to May_1–31_)					
Type					
Intercept	3.1	0.12	-	-	-
Miniature donkeys	0.03	0.08	0.7	−0.12	0.19
Hybrids	−0.32	0.07	0.000004	−0.46	−0.18

Period_mid Aug–late Oct_: ID variance = 0.10 (SD = 0.32), Month variance = 0.02 (SD = 0.13), State variance = 0.0000000004 (SD = 0.00002), Residual variance = 0.18 (SD = 0.42). Period_early Nov–early Aug_: ID variance = 0.10 (SD = 0.17), Month variance = 0.17 (SD = 0.41), State variance = 0.0000000004 (SD = 0.00002), Residual variance = 0.28 (SD = 0.53).

## Data Availability

Data supporting the reported results can be found here: https://github.com/IvanekLab/Donkey-and-hybrid-ACTH (accessed on 10 December 2025).

## Supplementary Materials

The following supporting information can be downloaded at https://www.mdpi.com/article/10.3390/ani16020290/s1. Figure S1: Evaluation of model assumptions for the multivariable linear mixed-effects model, including histograms for assessing normality and scatterplots for examining residual homoscedasticity during (top) Period_mid Aug-late Oct_ and (bottom) Period _early Nov-early Aug_; Table S1: Upper reference limits for adrenocorticotropin hormone (ACTH) concentrations estimated from Box-Cox-transformed data of apparently healthy hybrids. Values were calculated using a bootstrapped robust method.

## Abbreviations

The following abbreviations are used in this manuscript:

US	United States
ACTH	Adrenocorticotropic Hormone
PPID	Pituitary Pars Intermedia Dysfunction
CLSI	Clinical Laboratory and Standards Institute
CI	Confidence Interval
