# Supplementary material for: Both Season and Equid Type Affect Endogenous Adrenocorticotropic Hormone Concentrations in Healthy Donkeys, Mules and Hinnies in the United States
Source: Animals (Basel). 2026 Jan 16;16(2):290. doi: 10.3390/ani16020290 (PMC12837305; doi:10.3390/ani16020290)
Supplement: Supplementary file 1 [file animals-16-00290-s001.zip › animals-4070193-supplementary.pdf]

# Both Season and Equid Type Affect Endogenous Adrenocorticotropic Hormone Concentrations in Healthy Donkeys, Mules and Hinnies in the United States

Supplementary Information

Erin L. Goodrich [1,*], Sebastián Gonzalo Llanos-Soto [1], Renata Ivanek [1], Toby Pinn-Woodcock [1], Elisha Frye [1], Amy Wells [1], Stephen R. Purdy [2], Emily Berryhill [3] and Ned J. Place [1]

[1] Department of Population Medicine and Diagnostic Sciences, College of Veterinary Medicine, Cornell University, Ithaca, NY 14850, USA; sgl67@cornell.edu (S.G.L.-S.); ri25@cornell.edu (R.I.); tlp52@cornell.edu (T.P.-W.); eab73@cornell.edu (E.F.); amywells@cornell.edu (A.W.); njp27@cornell.edu (N.J.P.)

[2] Private Veterinary Practice and Nunoa Project Peru, Belchertown, MA 01007, USA; nunoavet@gmail.com

[3] Department of Medicine and Epidemiology, School of Veterinary Medicine, University of California, Davis, CA 95616, USA; ehberryhill@ucdavis.edu

* Correspondence: elg25@cornell.edu

Table of contents

Supplementary Table S1. Upper reference limits for adrenocorticotropin hormone (ACTH) concentrations estimated from Box-Cox-transformed data of apparently healthy hybrids. Values were calculated using a bootstrapped robust method. 3

Supplementary Figure S1. Evaluation of model assumptions for the multivariable linear mixed-effects model, including histograms for assessing normality and scatterplots for examining residual homoscedasticity during (top) Period $_{\text{mid Aug-late Oct}}$ and (bottom) Period $_{\text{early Nov-early Aug}}$. 4

Supplementary Table S1. Upper reference limits for adrenocorticotropin hormone (ACTH) concentrations estimated from Box-Cox-transformed data of apparently healthy hybrids. Values were calculated using a bootstrapped robust method.

	Jun_{1-15}	Jun_{16-30}	$July_{1-15}$	Jul_{16-31}	Aug_{1-15}	Aug_{16-31}	Sep_{1-15}	Sep_{16-30}	Oct_{1-15}	Oct_{16-31}	Nov_{1-15}	Nov_{16-30}	Dec_{1-31}	Jan_{1-31}	Feb_{1-29}	Mar_{1-31}	Apr_{1-30}	May_{1-31}
**Hybrids**																		
Sample size	ND	28	28	28	28[a]	28	28	28	28	28	ND	28	28	28	28	28	27	ND
Upper reference limit	ND	33.8	65.5	29.8	53.5	204.4	236.2	184.4	204.4	133.7	ND	49.0	63.3	45.4	20.0	51.6	23.8	ND
90% CI lower	ND	23.4	40.8	26.6	34.9	121.8	134.4	97.2	139.3	94.9	ND	35.7	39.9	28.0	16.8	27.7	15.9	ND
90% CI upper	ND	50.8	107.5	32.9	74.5	412.1	703.6	ND[b]	315.6	177.1	ND	66.2	89.7	139.2	23.2	147.9	34.6	ND

90% CI= 90% confidence interval. [a]The data was found to be non-normally distributed after back-transformation following the Box-Cox transformation, based on the D'Agostino-Pearson test. ND = Insufficient data for estimation. [b]The presence of an individual presenting a very high ACTH concentration (1,054 pg/mL) prevents an accurate estimation of the upper bound of the 90% CI.

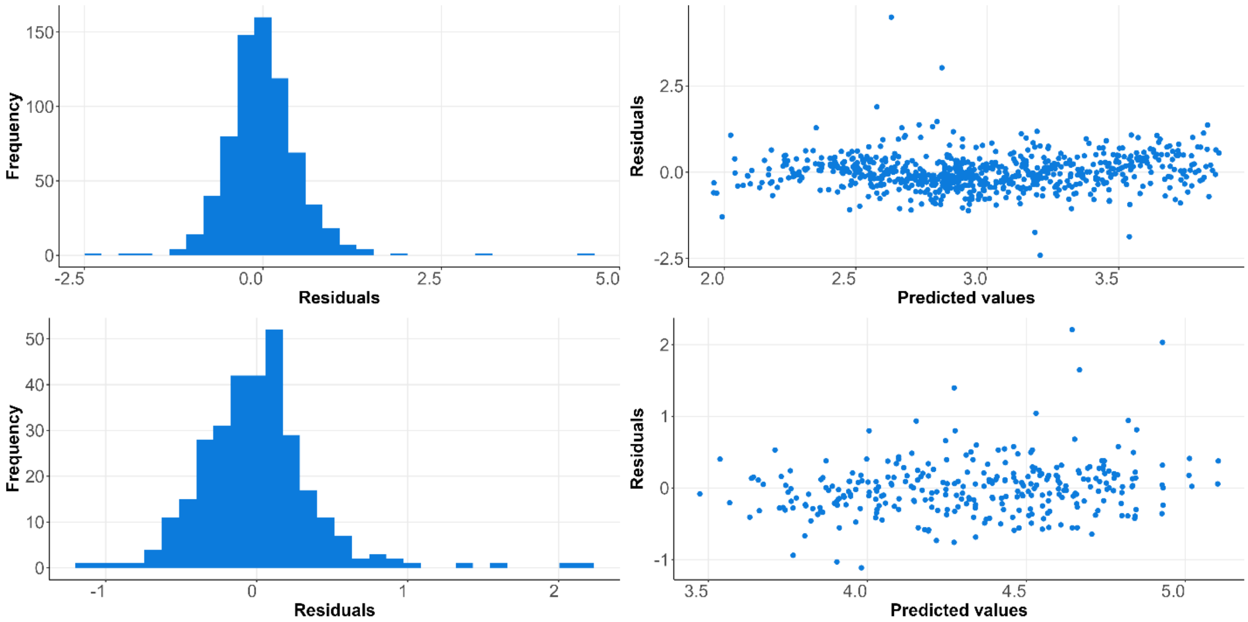

Supplementary Figure S1. Evaluation of model assumptions for the multivariable linear mixed-effects model, including histograms for assessing normality and scatterplots for examining residual homoscedasticity during (top) Period $_{\text{mid Aug-late Oct}}$ and (bottom) Period $_{\text{early Nov-early Aug}}$.
